# COVID‐19 was found in a patient's cerebrospinal fluid who presented with a severe form of Guillain‐Barre syndrome; A successful Sudanese story: Case report

**DOI:** 10.1002/ccr3.4597

**Published:** 2021-08-23

**Authors:** Etedal Ahmed A. Ibrahim, Khabab Abbasher Hussien Mohamed Ahmed, Elmuntasir Taha Salah, Mohammed Eltahier Abdalla Omer

**Affiliations:** ^1^ Faculty of Medicine Al Neelain University Khartoum Sudan; ^2^ The National Centre for Neurological Sciences Khartoum Sudan; ^3^ Faculty of Medicine University of Khartoum Khartoum Sudan; ^4^ Faculty of Medicine National Ribat University Khartoum Sudan; ^5^ Faculty of Medicine and Health Sciences Gadarif University Al Qadarif Sudan

**Keywords:** COVID‐19, Guillain‐Barre syndrome, neurology

## Abstract

COVID‐19 is a mysterious disease presented in different ways, so we have to deal with each patient nowadays thoroughly, including COVID‐19 testing as routine test. The Case report discusses the rare finding of COVID‐19 in CSF of GBS patient.

## Abstract 2

COVID‐19 is a mysterious disease presented in different ways, so we have to deal with each patient nowadays thoroughly, including COVID‐19 testing as routine test. The Case report discusses the rare finding of COVID‐19 in CSF of GBS patient

.

## Graphical abstract

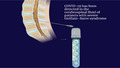

## 1 BACKGROUND

Neurological manifestation and complications are common due to COVID‐19. It affects higher functions, cranial nerves, and the motor system. The authors report a case of GBS as an example of a success story in managing a complicated case of COVID‐19 in an elderly man with signs of a poor prognosis.

The World Health Organization (WHO) was notified in December 2019 about COVID‐19, a new coronavirus detected in Wuhan, China, as the cause of an outbreak of a lower respiratory tract infection.^1^ The WHO then declared it a Public Health Emergency of International Concern on January 30, 2020. On February 11, 2020, the WHO announced a name for the new coronavirus disease: COVID‐19.^2^ To date, there are 8,860,331 cases of COVID‐19 globally including 465,740 deaths. The highest number of cases is in America at 4,370,519 with 4996 deaths. In the Eastern Mediterranean WHO region, there are 914,518 cases with 20,531 deaths. Sudan is a member of the Eastern Mediterranean WHO region and reported 8580 with 521 deaths.^3^ The virus mainly causes pneumonia and acute respiratory distress syndrome,^4^ as well as a multi‐organ disease affecting the kidneys, brain, heart, liver, and other organs.^5^ It leads to serious complications such as a cytokine storm, septic shock, blood clots, and immune‐mediated injuries.^6^, ^7^, ^8^ Neurological manifestations and complications are common due to COVID‐19. It affects higher functions, cranial nerves, and the motor system. It can lead to headaches, convulsions, mental and psychological changes, delirium, and insomnia. Guillain‐Barre syndrome can occur as a consequence of or in co‐incidence with COVID‐19, but it is very rare.^9^, ^10^, ^11^ The authors report a case of Guillain‐Barre syndrome as an example of a success story in managing a complicated case of COVID‐19 in an elderly man with signs of a poor prognosis.

## 2 CASE PRESENTATION

A previously healthy 70‐year‐old man (without hypertension or diabetes) presented (on the June 25, 2020) at the Emergency Department with complaints of lower limb weakness with an acute onset of numbness and the feeling of dead lower limbs preceded by a cough, which was dry and paroxysmal, accompanied by mild chest discomfort and a high‐grade fever without sweating or rigors. The fever and cough lasted for 7 days before the occurrence of weakness. His condition progressed over a day involving the upper limbs, neck, and facial muscles, and the patient was unable to turn in bed, stand, walk independently, move his upper limbs, or close his eyes. Difficulty swallowing, nasal regurgitation, or choking was not seen, and he had normal sensations and sphincters. Additionally, no convulsions, loss of consciousness, or other symptoms related to cranial nerves or higher functions were seen.

On examination, the patient was conscious, alert, and orientated to time, place, and person. A mini‐mental status examination (MMSE) was at 30. A cranial nerve examination revealed bilateral facial nerve palsy on the right side with facial deviation to the left, and the inability to close both eyes and blow his cheeks to whistle. Nystagmus, ophthalmoplegia, diplopia, cerebellar symptoms, and bulbar palsy were not detected. He had a normal jaw jerk with weak neck flexion. Furthermore, an upper limb examination showed hypotonia with absent reflexes and a muscle power assessment (MRC) was at grade 3 proximally and grade 2 distally, with normal sensations and absent tendon reflexes. A lower limb examination also revealed hypotonia with an MRC of grade 2 proximally and distally, absent reflexes, normal sensations, a flexor plantar response with normal coordination, and the patient was unable to walk.

**FIGURE 1:**
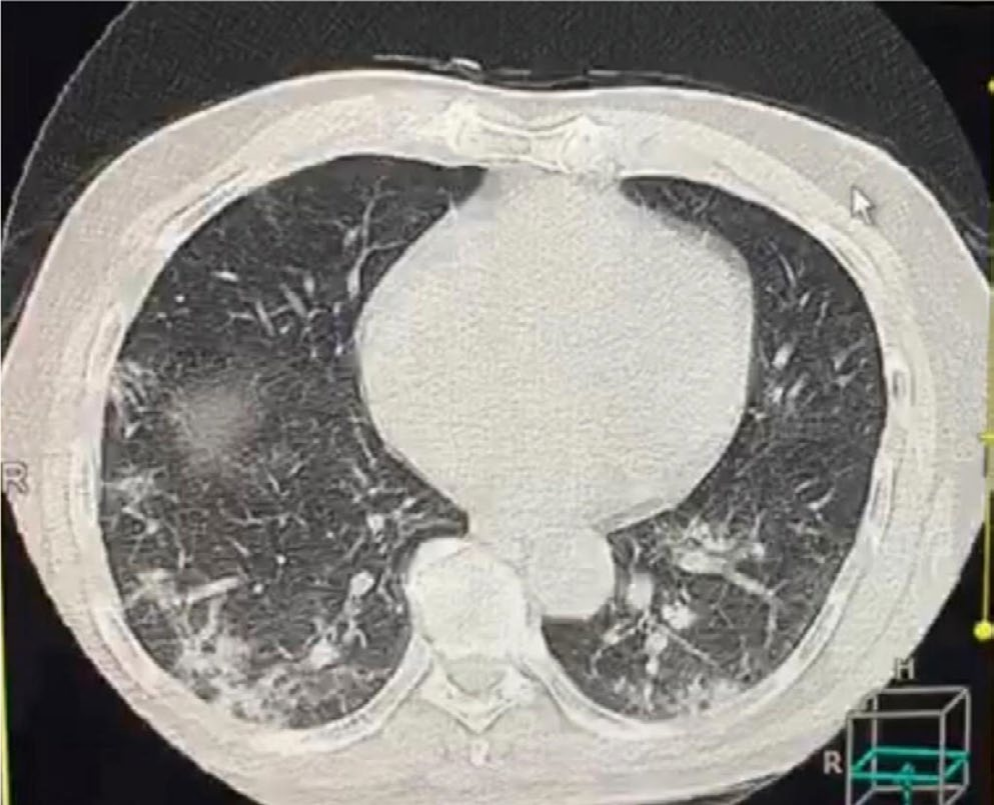
Computerized tomography of the chest of a 70‐year‐old man showing a ground‐glass appearance

**FIGURE 2:**
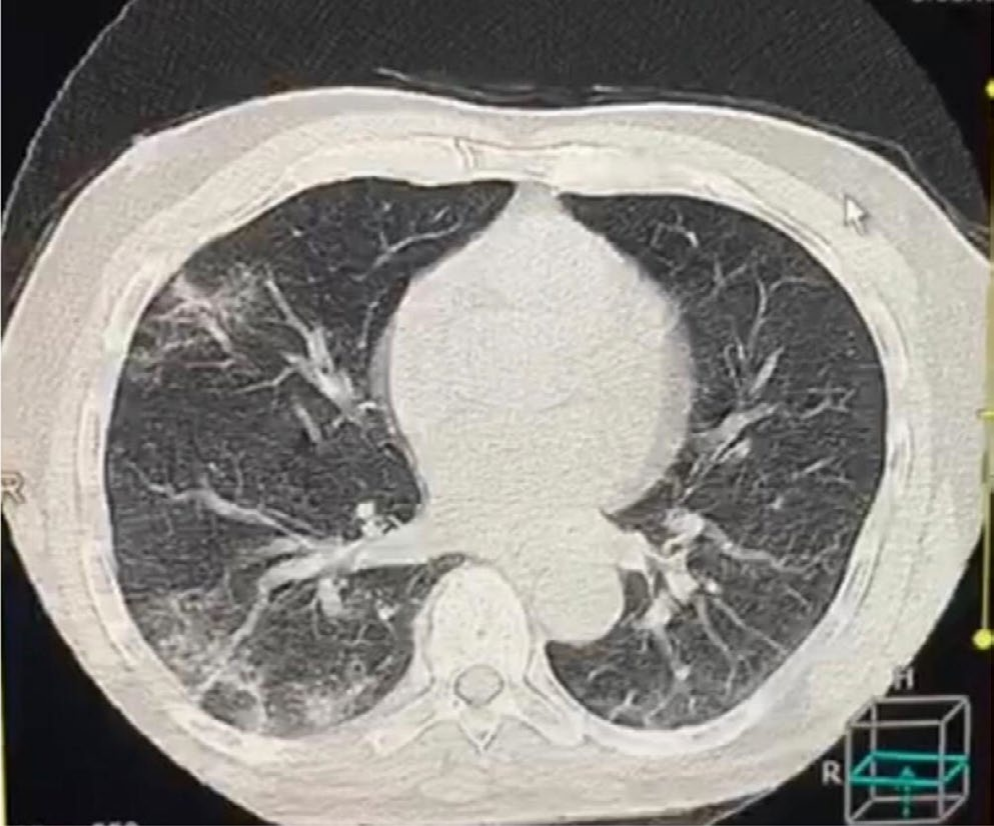
Computerized tomography of the chest of a 70‐year‐old man showing a ground‐glass appearance

General investigations were conducted with complete blood counts showing hemoglobin (Hb) 11 g, total white blood cells 6, lymphocytes 12%, C‐reactive protein (CRP) 110, erythrocyte sedimentation rate 70, platelets 396, serum ferritin 1000 ng/ml, blood urea 40 mg/dl, serum creatinine 0.9 mg/dl, serum potassium 3.5 mmol/L, sodium 135 mmol/L, alanine transferase 40, aspartate transaminase 20, alkaline phosphatase 150, random blood sugar 120 mg/dl, and a positive COVID‐19 test. A computerized tomography (CT) chest scan showed a ground‐glass appearance (Figures 1 and 2), and a nerve conduction study (NCS) reported demyelinating neuropathy consistent with acute inflammatory demyelinating polyradiculoneuropathy. Although it is a rare finding, a cerebrospinal fluid (CSF) examination was positive for both COVID‐19 and supporting the diagnosis of Guillain‐Barre syndrome.

## 3 DISCUSSION

Acute inflammatory demyelinating polyradiculoneuropathy or Guillain‐Barre syndrome is an immune‐mediated nerve disease. Reported causes of the syndrome are campylobacter, mycoplasma, influenza, Zika virus, cytomegalovirus, HIV, and lymphoma.^12^ Coronavirus (SARS‐COV 2) or COVID‐19 is a rare cause of Guillain‐Barre syndrome.^11^, ^12^, ^13^ There are very few cases worldwide with COVID‐19 causing GBS with some of these cases showing a good response to intravenous immunoglobulin.^14^ Other cases showed axonal neuropathy in the NCS, while others showed demyelinating neuropathy which is a common type in North America and Europe but thought to be rare in Africa. Other types of GBS according to the NCS classifications are acute motor axonal neuropathy (AMAN) and acute sensory‐motor neuropathy (ASMAN) which are more frequent in China, Japan, and Mexico, and Miller Fisher syndrome (MFS) which is more common in Asia.^15^In Sudan, we have mixed types of AIDP, AMAN, ASMAN, and MFS.^16^ In this case, the patient first presented with weakness ascending in nature involving the upper limbs, neck, and facial muscles on the same day, preceded by a high‐grade fever with rigor and sweating, a dry cough, soreness, and chest discomfort with normal sensations, sphincter, and flexor plantar responses. The patient came to the Emergency Department at the National Centre for Neurological Sciences in Khartoum with signs suggestive of COVID‐19 infection‐causing GBS. A patient workup was conducted including general investigations and complete blood counts which showed lymphopenia, high CRP and serum ferritin levels, normal arterial blood gases and the presence of a ground‐glass appearance which is highly suggestive of COVID‐19 in conjunction with the symptoms. A nasal swab was taken and sent to the laboratory. After that, treatment with intravenous immunoglobulin was started in doses of 28 g per day. While the nasal swab result was pending, the patient showed immediate improvement after IVIG; the power changed from MRC grade 3 to MRC grade 2. Moreover, the patient received supportive management for COVID‐19 in the form of paracetamol and vitamins. The patient reported that he was satisfied with the outstanding response to the treatment. A nerve conduction study showed a decrease in conduction velocity and delayed latencies with a dispersed response. This was due to the presence of demyelination, which is suggestive of the diagnosis of acute inflammatory demyelinating polyradiculoneuropathy or GBS. A follow‐up with the patient after 1 month showed complete recovery, the patient walking without support.

## 4 CONCLUSION

Patients with COVID‐19 can present with any symptoms, including diseases of the nervous system and peripheral nerves such as Guillain‐Barre syndrome, which sometimes responds to IVIG treatment. In this case, there was an excellent response despite the poor prognostic factors such as old age, gender, rapid onset of complete paralysis, lymphopenia, high inflammatory markers, and a ground‐glass appearance on a CT chest scan. The presence of the virus can be seen in the CSF, which existed in this case.

## CONFLICT OF INTEREST

The authors have no conflict of interest to declare.

## AUTHOR CONTRIBUTIONS

EIA served as the first author, collected the data, analyzed the results, and wrote the manuscript. KH and MEO served as the second and fourth authors, wrote the manuscript, revised the manuscript, and did editing. EST served as the third author, collected and analyzed the data. All authors read and approved the final manuscript.

## ETHICAL APPROVAL

Not applicable.

## CONSENT TO PARTICIPATE

Verbal and written consents were obtained from the patient before writing the case or using investigations.

## CONSENT FOR PUBLICATION

Written consent to publish this information was obtained from the patient. The patient gave written consent for his personal clinical details along with his CT chest images to be published in this study. This patient has not been reported in any other submission by the authors or anyone else.

## Data Availability

The datasets used and/or analyzed during the current study are available from the corresponding author on reasonable request.
